# Editorial: Extreme Eating Behaviours

**DOI:** 10.3389/fpsyt.2020.639219

**Published:** 2021-01-25

**Authors:** Hubertus Himmerich, Ahmad Saedisomeolia, Ute Krügel

**Affiliations:** ^1^Department of Psychological Medicine, King's College London, London, United Kingdom; ^2^School of Nutritional Sciences and Dietetics, Tehran University of Medical Sciences, Tehran, Iran; ^3^Medical Faculty, Rudolf Boehm Institute of Pharmacology and Toxicology, University of Leipzig, Leipzig, Germany

**Keywords:** eating disorders, anorexia nervosa, bulimia nervosa, binge eating disorder, obesity

## Importance of Extreme Eating Behaviors

Extreme eating behaviors are an increasing global health threat, as their prevalence is rising (1). If we take a look at both ends of the spectrum, ~650 million people are obese according to the World Health Organization (WHO) (2), and anorexia nervosa (AN) is one of the most common chronic disorders in adolescence. It is also the most lethal psychiatric disorder with a risk of death that is five times higher compared to people of the same age and gender without AN (3, 4).

In recent decades, our diagnostic knowledge of various eating disorders (EDs) has increased. These disorders include AN, bulimia nervosa (BN), binge eating disorder (BED), avoidant-restrictive food intake disorder (ARFID), pica and rumination. The latest edition of the Diagnostic and Statistical Manual of Mental Disorders (DSM-5) provides clinically useful and distinct diagnostic criteria for these disorders (5). Nonetheless, treatment options are still limited. For example, the only psychopharmacological treatment options for all EDs approved in some countries are fluoxetine for BN and lisdexamfetamine for BED (6).

We are currently increasing our understanding of how obesity and EDs are associated with changes in the composition of gut microbiota, in the intake of macro- or micro-nutrients, the immune, endocrine and nervous systems, and how these changes lead to physical and mental health consequences (7–10). However, the treatment outcome for EDs and their physical and mental health consequences is still unsatisfactory. A recent cohort study showed that only 30% of patients with AN recovered after 9 years (11).

This special collection is dedicated to disorders associated with extreme eating behaviors—the EDs and obesity. It consists of a variety of articles and study types including a historical study, an animal study, a case series, a feasibility study, cross-sectional and longitudinal clinical studies, surveys, meta-analyses, and systematic reviews; and it addresses historical, social, psychological, and biological aspects as well as symptoms and therapeutic options for extreme eating behaviors.

## Content of the Special Collection

### Historical Aspects of Extreme Eating Behaviors

Bergner et al. performed an in-depth historical review of the most significant German-language psychiatric textbooks throughout the past 200 years, regarding ED diagnoses and descriptions of disordered eating behavior. Interestingly, the authors found that nineteenth and early twentieth century psychiatrists such as Kraepelin, Bumke, Hoff, Bleuler, and Jaspers reported symptom clusters of extreme eating behavior such as food refusal and vomiting which show striking similarities to the description of specific eating disorder subtypes in current diagnostic manuals such as DSM-5. However, these historic psychiatrists partly classed those behavioral symptom clusters as features of diagnoses which are no longer used by the majority of psychiatrists, such as neurasthenia, hypochondria, and hysteria (Bergner et al.).

### Social and Psychological Aspects of Extreme Eating Behaviors

Five articles in this special collection focus on significant social and psychological factors of extreme eating: culture change, executive functions, temporal discounting, loss aversion, and Theory of Mind (ToM).

Shekriladze et al. conducted a survey on culture change and eating patterns, which showed that moving to Western countries (UK, USA) increased dietary restriction among Georgian women. They concluded that integration problems in the new host culture predict elevated eating, shape and weight concerns among women and that acculturation conditions may be linked with integration and well-being outcomes.

Gisbert Cury et al. conducted a systematic review and meta-analysis on executive functions in BED and ascertained that, based on current scientific literature, BED patients had worse performance on working memory tasks compared to obese individuals without BED. Kekic et al. reported data derived from an online survey that examined temporal discounting, which is the tendency to act on immediate pleasure-driven desires, due to the devaluation of future rewards. Their data revealed that temporal discounting correlated with the frequency of compulsive overeating, food addiction, ED psychopathology, and body mass index (BMI). Sagiv et al. performed a clinical study which revealed that loss aversion scores were lower among participants with EDs (AN, BN) compared to healthy controls and that this feature was related to non-suicidal self-injury and suicidal ideations. Sedgewick et al. focused on ToM in a cross-sectional clinical study involving healthy controls and patients with AN with and without autistic features. Interestingly, they did not find any quantifiable ToM difference between these groups as one would have expected from autism studies in people without AN. Studies in people with an autism spectrum disorder but without an ED usually indicate difficulties with ToM.

### Biological Aspects of Extreme Eating Behaviors

Obesity and AN are the two ends of the spectrum of extreme eating behaviors with manifold biological causes including genetic factors, the microbiome, metabolic and endocrine mechanisms, the immune system, and the brain, which are influenced by environmental and nutritional factors (12). We received three articles mainly concerned with the immune and the endocrine systems. Schmidt et al. conducted a longitudinal clinical study in normal weight, overweight and obese people (BMI range: 18.3–61.4 kg/m^2^) on depressiveness, body weight, physical activity, and cytokine levels. They confirmed previous results from human (13) and animal studies (14, 15) showing that depressiveness was positively associated with certain pro-inflammatory cytokine levels. Furthermore, physical activity was negatively associated with interleukin (IL)-4, IL-5, IL-10, granulocyte-macrophage colony-stimulating factor (GM-CSF), interferon (IFN)-γ, and tumor necrosis factor (TNF)-α; and BMI predicted IL-12 and IL-13 levels. Stanikova et al. reported data from an epidemiological cohort study that included 3,124 adult women. They found higher testosterone levels in obese vs. normal-weight women and in women with vs. women without anxiety symptomatology. Tyszkiewicz-Nwafor et al. looked at growth factors and hormonal changes in people with AN in a longitudinal clinical study. In their sample, brain-derived neurotrophic factor (BDNF) serum levels were decreased in malnourished AN patients, which normalized with weight recovery. Oxytocin serum levels, however, were increased in malnourished AN patients and did not normalize with partial weight recovery.

### Symptoms Related to EDs and Obesity

Baldofski et al. analyzed data from two large European multi-center studies: MooDFOOD and NESDA. In their sample of more than 500 people, somatic and vegetative depressive symptoms such as pain, changes in appetite and weight, gastro-intestinal symptoms and arousal-related symptoms were associated with both a higher BMI and a higher waist-to-hip ratio (WHR). In a clinical study by Minkwitz et al., subjective sleepiness did not differ much between obese and non-obese people, whereas depressed and non-depressed people differed significantly with regard to subjective sleepiness. Objective sleepiness measures, however, did not differ significantly between people with obesity, people with depression, people with both obesity and depression, and healthy controls.

A systematic review and quantitative analysis by Riedlinger et al. deduced that gastrointestinal (GI) symptoms and impaired gastric transit are frequent features of EDs and that serious GI complications such as gastric dilatation have been observed. Stein et al. provided a case series describing risk-taking behavior in four female patients with long-standing binge-purge type AN. The description of these patients and their history, course of the disease and risky behaviors expound important environmental vulnerabilities, purging behavior as well as other impulsive and non-impulsive comorbidities.

### Therapies for EDs and Obesity

This special collection on extreme eating behaviors also sheds light on experimental and potential future treatment strategies. Kan et al. contributed a systematic review and meta-analysis of dropout and metabolic effects of antipsychotics used in AN. In their analysis, drug-related factors, such as side effects, played a lesser role than personal reasons for the discontinuation of antipsychotic treatment under trial conditions. This highlights the importance of patients' personal motivation for drug treatment in AN. Lu et al. reported in an experimental animal study that electroacupuncture reduced food intake and body weight, and improved laboratory parameters in obese mice. Thus, electroacupuncture might be an innovative approach to treat extreme eating behaviors and their health consequences. An innovative psychotherapeutic approach was presented by Rudolph and Hilbert. Their clinical pilot study tested a short-term cognitive behavior therapy (CBT) approach, which was based on manuals for BED and depressive disorders. This treatment for patients following bariatric surgery led to a significant reduction of body weight, improvement of ED psychopathology, depressive symptoms, and self-esteem.

## Thanks to All Authors, Reviewers, Editors and Frontiers

The articles in this Research Topic provide a comprehensive, cutting-edge and inspiring view on research and clinical practice in the field of extreme eating behaviors. We would like to express our thanks to all authors for their hard work, excellent manuscripts, and for submitting work to this special collection. The call attracted authors from Australia, Brazil, China, Georgia, Germany, Ireland, Israel, the Netherlands, Poland, Slovakia, Spain, Sweden, and the United Kingdom. We also thank all the reviewers and editors involved in the preparation of this special issue as well as the publisher Frontiers and their team for always being supportive. We hope that this special collection presents a useful and appealing overview of current knowledge about extreme eating behaviors, which will reach many readers and inspire future research.

## Author Contributions

All authors drafted the manuscript together, discussed it, and approved its final version.

## Conflict of Interest

The authors declare that the research was conducted in the absence of any commercial or financial relationships that could be construed as a potential conflict of interest.
